# CAR-T Cells/-NK Cells in Cancer Immunotherapy and the Potential of MSC to Enhance Its Efficacy: A Review

**DOI:** 10.3390/biomedicines10040804

**Published:** 2022-03-30

**Authors:** Ler Yie Chan, Sylvia Annabel Dass, Gee Jun Tye, Siti A. M. Imran, Wan Safwani Wan Kamarul Zaman, Fazlina Nordin

**Affiliations:** 1 Centre for Tissue Engineering and Regenerative Medicine (CTERM), Faculty of Medicine, Universiti Kebangsaan Malaysia, Jalan Yaacob Latiff, Bandar Tun Razak, Kuala Lumpur 56000, Malaysia; chanleryie13@gmail.com (L.Y.C.); siti.imran@ukm.edu.my (S.A.M.I.); 2 INTEC Education College, Jalan Senangin Satu 17/2A, Seksyen 17, Shah Alam 40200, Malaysia; 3 Institute for Research in Molecular Medicine (INFORMM), Universiti Sains Malaysia, Minden 11800, Malaysia; sylviadass@usm.my (S.A.D.); geejun@usm.my (G.J.T.); 4 Department of Biomedical Engineering, Faculty of Engineering, Universiti Malaya, Kuala Lumpur 50603, Malaysia; wansafwani@um.edu.my; 5 Centre for Innovation in Medical Engineering (CIME), Department of Biomedical Engineering, Faculty of Engineering, Universiti Malaya, Kuala Lumpur 50603, Malaysia

**Keywords:** CAR, chimeric antigen receptor, CAR-T cell, CAR-NK cell, cancer, immunotherapy, MSC, mesenchymal stem cell

## Abstract

The chimeric antigen receptor (CAR) plays a dynamic role in targeting tumour-associated antigens in cancer cells. This novel therapeutic discovery combines fragments of monoclonal antibodies with the signalling and co-stimulatory domains that have been modified to its current fourth generation. CAR has been widely implemented in T-cells and natural killer (NK) cells immunotherapy. The significant advancement in CAR technology is evident based on numerous ongoing clinical trials on CAR-T/-NK cells and successful CAR-related products such as Kymriah (Novartis) and Yescarta (Kite Pharma, Gilead). Another important cell-based therapy is the engineering of mesenchymal stem cells (MSC). Researchers have been exploring MSCs and their innate homing abilities to tumour sites and secretion cytokines that bridge both CAR and MSC technologies as a therapeutic agent. This combination allows for both therapies to overcome each one’s flaw as an immunotherapy intervention. Herein, we have provided a concise review on the background of CAR and its applications in different cancers, as well as MSCs’ unique ability as delivery vectors for cancer therapy and the possibility of enhancing the CAR-immune cells’ activity. Hence, we have highlighted throughout this review the synergistic effects of both interventions.

## 1. Introduction

Cellular immunotherapy, also known as adoptive cell therapy, has shown significant progress and advances utilising engineered immune cells to eliminate cancer. A notable contributor to immunotherapy is the expression of chimeric antigen receptors (CAR) on the surface of immune cells, mostly T-cells and natural killer (NK) cells. This form of cancer treatment involves the use of “living drugs” [1] because the patient’s cells or cells from donors are genetically engineered and processed for cancer treatment. Hence, the specificity and activity of the engineered immune cell are directed, in this case, towards tumour cells [2]. CAR-T cell therapy has been studied extensively over the years, and currently, there are approximately 1000 clinical trials. CAR-NK cell therapy has also attracted a great deal of interest in recent years to overcome the limitations of CAR-T cells. However, CAR therapies may have limited therapeutic potential, especially in solid malignancies (toxicity, escape of antigen, limited stability, etc.). This has paved the way for more studies to improve the efficacy of CAR immune cells, and one of the methods is the introduction of mesenchymal stem cells (MSC) as biological vehicles [3,4,5,6,7]. A biological molecule delivery system may sustain the anti-tumour response of CAR immune cells [8]. This review discusses the application of CAR-T and CAR-NK cells in immunotherapy, the role of MSCs in CAR treatment, the efficacy of CAR-T and CAR-NK cell therapies in solid and non-solid tumours, their limitations and advances made in the CAR structure and how modified MSCs can improve CAR-T/NK cell treatment. Current preclinical and clinical trials associated with CAR-T/NK cell therapy and MSC-assisted CAR treatments are reviewed in this article.

## 2. Chimeric Antigen Receptor (CAR)

The CAR protein is composed of two domains: (a) The extracellular tumour-antigen receptor that specifically recognises tumour-associated antigens (TAA) on the cell-surface membrane of cancer cells (e.g., CD19 on B-cells); and (b) the intracellular signal transduction domain, which stimulates the engineered cell’s proliferation and function [1,9]. The extracellular domain is the antigen-binding site of monoclonal antibodies (scFv, sdab, etc.), while the intracellular domain is a combination of natural TCR complex and co-stimulatory molecules [1,9,10]. Modifications made to the intracellular signal transduction domain give rise to several generations of CARs [10]. The design of CARs to treat cancer relies on specific TAA while the signalling and co-stimulatory domains depend on the immune cell used [11]. The expression of CARs on cell surfaces relies on gene transfer technology, mainly viral-based gene transfer. This includes the use of alpha-retroviruses, gamma-retroviruses and lentiviruses for gene engineering. Non-viral methods involve DNA-based transposons or the direct transfer of mRNA by electroporation [12]. We will discuss two of the most common CAR-immune cells involved in cancer treatment, including a brief description of the therapeutic mechanism of each intrinsic immune cell, their sources and the production process of the CAR-immune cells. It is also important to note that genetically engineered immune cells carry out the same cytotoxic mechanisms as unmodified immune cells. The presence of CARs facilitates the binding to specific TAA in order for cytotoxic activities to be directed to the respective tumour [11].

### 2.1. CAR-T Cells

T-cells are responsible for the body’s cell-mediated immune responses and play a significant role in identifying antigens of tumour cells, proliferating to a great number and performing cytotoxicity upon appropriate signals. However, the genetic instability in tumour cells causes these cells to not have the required immunogenic markers for T-cell recognition. Mutations in major histocompatibility complex (MHC) genes, the immunosuppressive microenvironment and the expression of negative co-stimulatory molecules disrupt intrinsic T-cell antitumour activity. Prior to the development of CAR-T cells, researchers developed T-cells that expressed tumour-specific TCRs [13]. However, TCR-engineered T-cells have limited modifications in their cellular and molecular mechanism, hence this treatment is still subjected to human leukocyte antigen restriction, the negative regulatory factors of the tumour microenvironment and requires sufficient expression of MHC [10,13]. Consequently, the limitations of this method led to the development of genetically engineered T-cells expressing the recombinant receptor CAR in place of TCR. This enables specific targeting of cancer antigens and is MHC-independent [9,13].

T-cells that are genetically engineered to express CARs are often obtained from the peripheral blood of the patient via leukapheresis or phlebotomy followed by apheresis [13]. The CAR gene is transduced into the T-cells and the expansion of T-cells is observed ex vivo [10,13]. Purification is carried out, and the validation of its quality and sterility is required [14]. Lymphodepletion is carried out in patients prior to CAR-T cells administration [13].

Treatment of haematological malignancies using CAR-T cell therapy has shown significant achievements over the years. However, there are still challenges that need to be resolved before it can be used as a dominant therapeutic choice in cancer treatment. Other concerns such as its serious side effects should also be highlighted and resolved. Some known side effects are cytokine release syndrome (CRS) [15,16,17], immune effector cell-associated neurotoxicity syndrome (ICANS) [18,19], cytopenia [20,21], tumour lysis syndrome and off-tumour on-target toxicity [9,22,23].

### 2.2. CAR-NK Cells

Alternatively, research on genetically engineered natural killer (NK) cells that express CARs is of great interest because CAR-T cell therapy has its known limitations, such as suppression by regulatory T-cells (Tregs) [24]. It is also suggested that CAR-NK cells may be more beneficial than CAR-T cells as they are not suppressed by Tregs. When NK cells are activated, they can carry out cytotoxic activities without prior tumour antigen priming, which is essential for T-cell activation [24,25]. They release cytotoxic granules of perforin and granzyme, which causes target cell lysis [26,27]. In addition, NK cells also release tumour necrosis factor molecules (TNF), which increase death-ligand expression (TRAIL/FasL) on the NK cell surface that can be recognised by death receptors of tumour cells and initiate the caspase pathway of cell apoptosis [24,26,28]. Furthermore, NK cells secrete interferon-gamma (IFN-γ), which causes the recruitment of other immune effectors such as macrophages and dendritic cells to carry out alternative anti-tumour activities [27,29]. Another immune response of NK cells is its ability to kill cancers cells by antibody-dependent cellular cytotoxicity (ADCC). CD16 plays a key role in ADCC [27]. It recognises and binds to the Fc fragment of immunoglobulin G (IgG) antibodies opsonised on the surface of tumour cells [27,30]. An advantage of NK cells over T-cells is that they do not stimulate graft-versus-host disease (GVHD). Their allogeneic behaviour gives them potential as off-the-shelf therapies in the near future [31]. NK cells also do not cause severe toxicities such as CRS and ICANS [32]. The efficient intrinsic abilities of NK cells aptly complement suitable CAR structures.

**Figure 1:**
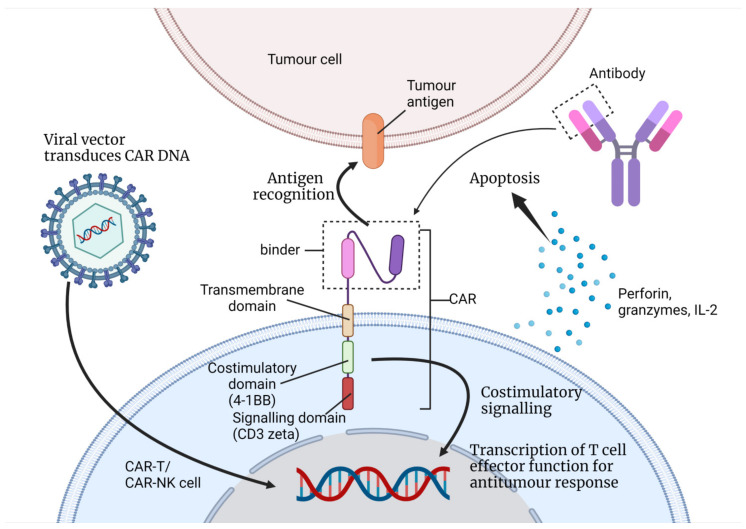
This figure illustrates how a T-cell/NK cell can be engineered to express CAR with its binder from monoclonal antibodies (single-chain variable fragment in the figure) as the antigen-binding receptor. This is a second-generation CAR with its single co-stimulatory domain (4-1BB or CD28) and its signalling domain (CD3zeta) [13]. A viral vector is used to transfer the DNA that codes for CAR into the nucleus of the immune cell. When the CAR receptor recognises the tumour antigen, the signal is amplified and transferred to the nucleus. This initiates a series of antitumour responses; the immune cell may proliferate and secrete cytokines, perforins, etc. (Created with BioRender.com).

The initial CAR structures used in engineered NK cells were similar to the CARs that were designed for T-cell-based therapy (Figure 1) [27]. Consequently, the CAR structures lacked specificity for NK cells as these CARs were tailormade for T-cells. Therefore, their signalling and co-stimulatory domains were more optimum for the T lymphocyte signalling pathways including the CD3zeta (signalling domain) and 4-1BB or CD28 (co-stimulatory domain). It is necessary to investigate the co-stimulatory domains that are better equipped and more specific for NK cell signalling in order to maximise its efficacy [24,27]. NK-specific co-stimulatory domains such as DNAX-activation protein 10 (DAP10), DAP12 and 2B4 showed greater cytotoxicity and increased IFN-γ secretion [27]. Moreover, CAR-NK cells are incorporated with inducible caspase 9 (iCas9) to eliminate the CAR-NK cells after their antitumour activities [33]. NK cells from the peripheral blood of donors, human NK92 cell lines, umbilical cord blood and induced pluripotent stem cells (iPSC) are suitable for the generation of CAR-NK, with each source having its advantages and limitations [27].

The production process of CAR-NK cells is rather similar to that of CAR-T cells. However, CAR-NK cells require further expansion and activation prior to infusion. It is necessary to produce CAR-NK cells that are homogenous, GMP-compliant and show a similar maturity stage. Common approaches include incubation with feeder cells, for example, the Wilms tumour cell line, irradiated K562 cells, or human B-lymphoblastoid cell line 721.221 [27]. Overall, the differences between CAR-T and CAR-NK cells are summarised in Table 1, while the similarities between the two CAR cells are discussed in Table 2.

## 3. Mesenchymal Stem Cells (MSC)

CAR-engineered T- and NK cells are potential frontiers of cancer treatment, but they are far from perfect. They suffer from exhaustion of T-cells and poor performance in solid tumours leading to the need for improving its efficacy [34]. An approach to improve them that has attracted the interest of the scientific community is the use of mesenchymal stem cells (MSCs are stromal cells that can self-renew and show multilineage differentiation [35]. They are adult stem cells isolated from a variety of sources such as bone marrow, adipose tissue, umbilical cord tissue or amniotic fluid. They are multipotent stem cells, hence they have the ability to differentiate into different types of stromal cells [36] allowing them to make and repair skeletal tissues, such as cartilage, bone and fat found in the bone marrow. MSCs also secrete a variety of chemokines and cytokines making them an attractive complement to CAR-T/NK therapy [8]. Initiating an acute immune response from the cytokine environment is thought to be able to sustain CAR-T cell activity. Due to CAR-T cells showing less capability in solid tumour elimination, MSCs are used to improve their efficiency. MSCs initiate and sustain an inflammatory environment that promotes tumour progression in cancer cells [37]. However, MSC’s tumour homing ability together with rapid engineering by viral vectors enables MSCs to migrate into tumour tissue [38]. Hence, MSCs can be used as vehicles to deliver therapeutic products such as biologically active proteins (supportive cytokines) and transgenic immune modulators to tumour tissue [8,38]. This causes the tumour bed to shift to an immune stimulatory environment from its initial inhibitory state [38]. Furthermore, human MSCs are also allogeneic, hence the cells can be used as off-the-shelf cell products [8]. A study by A.A Hombach et al. elaborated on the ability of the engineered MSCs to release both IL7 and IL12 that promoted homeostatic expansion and Th1 polarisation. Besides, the modified MSCs also showed more significant results in supporting CAR-T cell activity and improved the anti-tumour attack in a transplant tumour model compared to unmodified MSCs. As a result, CAR-T cell response is expected to be sustained and prolonged, which improves solid tumour lesions [8].

## 4. Application of CAR-T/CAR-NK Cells in Immunotherapy

### Current Clinical Trials Involving CAR-T/CAR-NK Cells

CAR-T cell therapy has garnered much attention over the past decade as a cancer therapy, especially in haematological malignancies. There are currently a total of 988 studies being conducted worldwide on CAR -T cell therapy for various cancers. The exponential increase in studies in recent years proves the significance of CAR-related therapies. A total of 943 studies were classified as interventional (clinical trials). The majority of studies are being conducted in East Asia, totalling 512 trials, followed by the United States with 372 ongoing studies and Europe with 89 trials.

The emergence of CAR-NK cell therapy as a subsequently promising therapeutic approach for cancer treatment has led to several studies of CAR-NK cell efficacy. However, the number of studies registered is relatively low as it is still relatively new. Only a total of 29 studies on CAR-NK cell therapy are currently being conducted worldwide, with the majority of trials being performed in China (15 studies), followed by the United States (7 studies), Australia (1 study) and Germany (1 study). Studies are also being conducted for alternative candidates of CAR modification, which includes iNKT cells (one trial in China), macrophages (one trial each in the United States and Europe) and gamma delta T-cells (one trial in China and one trial in Malaysia) (Data obtained from clinicaltrials.gov for trials registered by Q4 of 2021, accessed on 15 December 2021) [39].

CAR-T cell products that were approved to be used as drugs by the U.S. Food and Drug Administration (FDA) and European Commission (EC) from 2017 onwards are Kymriah (Novartis), Yescarta (Kite Pharma, Gilead), Tecartus (Kite Pharma, Gilead) and Breyanzi (Juno Therapeutics, Bristol-Myers Squibb Company) [40].

## 5. Efficacy of CAR-T/CAR-NK Cells in Both Solid and Non-Solid Tumours

### 5.1. CAR-T Cell Immunotherapy for Solid Tumours

Current CAR-T cell therapies are focused on highly specific tumour-associated antigens to prevent off target toxicities. Investigations into the efficacy of CAR-T cells on solid cancers were mainly conducted in animal models. The majority of antigen-specific CAR- cells show dose-dependent cytotoxicity; second- and third-generation CARs with co-stimulatory molecules enhance anti-tumour effects and improve efficacy; most but not all are able to eradicate cancer cells in xenograft mouse models. CAR-immune cell targets often overlap because these tumour-associated antigens are expressed on different tumours from different organs [41]. Some CAR-immune cell products have been translated for phase I/II clinical trials in human patients. The following paragraphs will give a brief overview of some clinical trials conducted on patients involving antigen-specific CAR immunotherapies.

The mesothelin antigen is overexpressed in several solid tumours such as malignant pleural mesothelioma, ovarian adenocarcinoma, pancreatic ductal adenocarcinoma and some lung cancers [42]. Haas et al. carried out a phase I study on mesothelin-specific CAR-T cells on patients with malignant pleural mesothelioma, ovarian adenocarcinoma and pancreatic ductal adenocarcinoma. A total of 15 patients were given a single infusion of CART-meso cells. Eleven patients achieved stable disease. Administration of cyclophosphamide pre-treatment did not improve persistence after 28 days despite improving the CAR-T cell expansion. From biopsied tissue, CART-meso DNA could be found. The CAR cells could expand and were well-tolerated but showed less clinical activity. Based on this study, subsequent research on mesothelin-specific CAR cells is currently ongoing [43].

The CD133 antigen is also overexpressed in 50% of hepatocellular carcinoma, pancreatic cancer, gastric cancer and intrahepatic cholangiocarcinoma cases. Wang et al. conducted a phase I clinical trial on 23 patients, 14 diagnosed with hepatocellular carcinoma, 7 with pancreatic carcinoma and 2 with colorectal carcinoma. The results show that 3 achieved partial remission, while 14 achieved stable disease. Twenty-one patients did not develop detectable de novo lesions, and biopsied tissue showed CD133-expressing cancer cells were eliminated after the infusions. The concern was the drop in haemoglobin and platelet count in almost all the patients, which eventually recovered within a week. In addition, bilirubinemia toxicity was also observed in three patients with an underlying illness. For long-term persistence of CAR-T cells, repeated infusion was given. Based on the results, CD133 CAR-T cells may prevent the occurrence of new metastasis, but additional studies would be required [44].

Goff et al. [45] conducted a pilot trial on EGFRvIII-specific CAR-T cells to treat recurrent glioblastoma in 18 enrolled patients. They used high cell doses, followed by a lymphodepleting regimen and administration of cytokine IL-2. Results show that the median progression-free survival was 1.3 months with an outlier at 12.5 months. The median overall survival was 6.9 months. However, two patients survived more than a year while one-third is still surviving after 59 months. However, this clinical trial was incapable of reaching the desired tumour regression and could not prolong the survival of patients with the disease [45].

### 5.2. CAR-NK Cell Immunotherapy for Solid Tumours

Xiao et al. [46] conducted a pilot study on RNA-engineered CAR-NK cells to treat metastatic colorectal cancer. NKG2D was fused to DAP12 by RNA electroporation to express CAR. Out of the three patients administered with the CAR-NK cells, two patients were given a low-dose intraperitoneal infusion of the CAR-NK cell and achieved a reduction in ascites and a decrease in the number of tumour cells in ascites samples. An ultrasound-guided percutaneous injection was given for the third patient with metastatic tumour sites in the liver, and rapid tumour regression was observed [46].

MUC1-specific CAR with the signalling domain CD28–CD137 and a truncated PD-1 peptide on the NK92 cell were able to lyse MUC1-positive tumour cells in vitro and in vivo. Thirteen patients with lung cancer, pancreatic cancer, colon cancer and ovarian cancer (PD-L1- and MUC1-positive) were infused with these cells. Three patients withdrew from the trial, nine patients achieved a stable disease while one patient showed progressive disease. No adverse cytokine storm or bone marrow suppression was observed. CAR-NK cell therapy was shown to have a stable clinical efficacy, mild side effects and ease of preparation [47].

An overview of the different target antigens and the efficacy of modified CAR-T/NK cells for some of the most common solid tumour cancers in the world for CAR-T and CAR-NK cell therapy are shown in Table 3.

### 5.3. CAR-T Cell Immunotherapy for Acute Lymphoblastic Leukaemia

CAR-immune cell therapy has shown significant results in the clinical trials of haematological malignancies treatment. Anti-CD19 CAR-T cells are the most effective in fatal relapsed/refractory B-cell acute lymphoblastic leukaemia (B-ALL) treatment [10,91]. Kymriah is an example of a CD-19-specific CAR-T cell approved by the FDA [9]. It is able to target and eliminate the CD-19-expressing B-lymphocytes, resulting in successful cancer remission in B-ALL patients [92]. Other potential targets include CD20 and the use of other immunoglobulin light chains [10]. The occurrence of antigen escape has spurred investigations into other targets such as CD22 and CD123 [10,93].

### 5.4. CAR-T Cell Immunotherapy for Chronic Lymphocytic Leukaemia (CLL)

Currently, it seems to be suggested that allogeneic stem cell transplantation is the preferred options in CLL [94]. CLL leads to early immune deficiency, hence limitations in expansion and proliferative response impairs CAR-T immunotherapy efficacy. A clinical trial using CD19-specific CAR-T cells did show feasibility and effectiveness with complete remission (CR) [95]. Another possible target for CLL CAR-T therapy is the tyrosine-protein kinase transmembrane receptor. The use of an anti-proliferative drug, ibrutinib, improves survival, engraftment and tumour clearance by CAR-T cells in xenograft models with concurrent infusions [96]. Clinical trials on CAR-T cell infusion after allo-HSCT were shown to be effective and safe in relapsed B-cell malignancies, hence the next phase of clinical trials would help establish its efficacy for CLL therapy [97].

### 5.5. CAR-T Cell Immunotherapy for Lymphomas

CAR-T cell has also shown great advancement in the treatment of relapsed or chemotherapy-refractory B-cell non-Hodgkin lymphoma. The ZUMA-1 trial suggested these patients receiving anti-CD19 CAR-T cell therapy have a manageable long-term safety profile with a durable response and median overall survival of more than two years [98]. CD20 and κ-light immunoglobulin are now well-established targets for treating NHL while CD30 is a new potential target for Hodgkin lymphoma [10].

### 5.6. CAR-T Cell Immunotherapy for Multiple Myeloma

Low expression of CD19 in multiple myeloma cells has led to the need of more specific targets [10]. It was shown that CD19 CAR-T cells were not only unable to eliminate the tumour cells but also damaged healthy cells [99]. A suitable target was found in the form of CD138. In a clinical trial by Guo et al., utilising anti-CD138 CAR-T cell therapy, most patients achieved stable disease for more than three months. One patient with advanced plasma cell leukaemia experienced a reduction in myeloma cells. No severe toxicities were observed, indicating its potential safety profile [100]. B-cell maturation antigen (BCMA)-specific CAR-T cells have also been intensely researched. In a case report utilising the BCMA-specific CAR-T cells, two non-responding patients who relapsed with refractory multiple myeloma achieved complete remission on the 30th day and persisted for more than 36 months [101].

### 5.7. CAR-NK Cell Immunotherapies for Haematological Malignancies

CAR-NK cells have been also intensively researched for their efficacy in haematological malignancies (Table 4). A CD-19-specific CAR-NK cells phase I/II clinical trial involving eleven patients with relapsed/refractory(R/R) CD19-positive cancers (NHL or CLL) showed no cytokine release syndrome, neurotoxicity or GvHD. Seven patients achieved CR, and the responses were rapid. The CAR-NK cells were also shown to persist in the patients’ blood for at least 12 months [32]. The efficacy and safety profile concluded from this trial is very promising for subsequent clinical studies. CAR-NK cells have advantages over CAR-T cells in treating T-lymphoid cancers as normal and malignant T-cells share the same antigens [24]. Myeloma cells also express several ligands for activating NK cells. Hence, CAR-NK cell immunotherapy has the potential to treat multiple myeloma [102].

## 6. Limitations of CAR-T/CAR-NK

CAR-T/NK immunotherapies have shown significant efficacy in haematological malignancies but have yet to successfully treat solid tumours. The limitations of CAR-T/NK cell therapy in solid tumours need to be overcome in order to enhance its therapeutic effects.

### 6.1. Limitations of CAR-T Cells

#### 6.1.1. Tumour Antigen Heterogeneity

A unique characteristic of solid tumours is the tumour’s antigen heterogeneity. This is a challenge for T-cells to detect cancer cells, hence, severely affecting CAR-T cell therapy. Furthermore, different cancer cells express a wide variety of tumour-associated antigens (TAA), which leads to difficulties in establishing targets for CAR engineering. Each antigen at different tumour sites is also expressed at a different level, and this affects the anti-tumour activity of CAR-T cells as they face difficulty in identifying specific antigens [1,103].

A method to overcome this adversity is by engineering T-cells to co-express multiple CARs. Other methods include programming CAR expression, temporarily adjusting target antigens, using various CAR-T cells in which each CAR is designed to target its specific antigen and using CARs that have two or more antigen recognition domains [104]. An alternative method is to target antigens that are overly expressed on the tumour cells, but this method would require an overly expressed antigen [44].

#### 6.1.2. Trafficking and Infiltration

Trafficking and infiltration of CAR-T cells into tumour tissue pose problems for solid tumour treatment. CAR-T cells are relatively more exposed to blood tumour or lymph tumour cells because they return to the bloodstream and lymphatic system. However, it is more challenging for CAR-T cells to penetrate solid tumour tissues through the vascular endothelium [5]. The expression of chemokines such as ligand-11 and 12 is lower in tumour cells, hampering the ability of CAR-T cells to migrate and penetrate tumour cells [105]. Furthermore, the dense fibrotic matrix of solid tumours and the development of various abnormalities pose challenges for the infiltration of CAR-T cells into the tumour tissue [1,106].

A suitable solution would be to carry out regional administration of CAR-T cells instead of a systemic administration. For example, intracranial delivery of CAR-T cells shows greater efficacy in glioblastoma treatment compared to intravenous infusion [86]. However, localised administrations are theoretically limited to single tumour lesions or oligometastatic disease [107]. It is important to improve or inhibit T-cell access to tumours. Aside from engineering T-cells to be responsive to specific chemokines of cancer cells, an alternative method would be to promote the release of chemokines that CAR-T cells can be responsive to. This is achieved via an oncolytic virus expressing CCl5 [1].

#### 6.1.3. Immunosuppressive Tumour Microenvironment

The immunosuppressive tumour microenvironment (TME) of solid tumours further limits the activity of CAR-T cells. Solid tumours provide a milieu for a range of immune suppressor cells that support tumour growth, angiogenesis and metastasis [108]. They release growth factors, local cytokines and chemokines such as VEGF, IL-4, IL-10 and TGFβ. Immune checkpoint molecules, CTLA-4 and PD-1, also interfere with antitumour immunity [1]. Furthermore, the competition between CAR-T cells and cancer cells as well as these immunosuppressor cells lead to a lack of oxygen and nutrients for CAR-T cells to function optimally [107]. The combination of immune-suppressing cells and molecules in the tumour microenvironment can lead to setbacks in CAR-T cell therapy.

The first solution is to modify T-cells to enhance their abilities in TME. T-cells that are engineered to express more potassium channels can reduce the suppression caused by potassium accumulation [109]. When administering CAR-T cells, it is necessary to destroy immunosuppressive cells that are in the way. This can be achieved by using suppressor antibodies that are genetically manipulated to weaken regulatory T-cells and myeloid-derived suppressor cells [1]. Moreover, cancer-associated fibroblasts can be destroyed by using fibroblast activation protein (FAP)-directed CAR-T cells. CAR-T cells that secrete extracellular matrix (ECM)-degrading enzymes can improve the penetration of CAR-T cells into tumour tissue [110]. The secretion of pro-inflammatory cytokines IL-12 not only activates CAR-T cell expansion and anti-tumour activity [111,112] but it also promotes macrophage recruitment and function. Checkpoint inhibitors targeting PD-1/PD-L1 are also potential solutions [1,113].

Another issue brought to light when it comes to CAR-T cell therapy is the various side effects that it is responsible for. These include cytokine release syndrome, tumour lysis syndrome, neurotoxicity, off-tumour on-target toxicity and oncogenic insertional mutagenesis [9].

### 6.2. Limitations of CAR-NK Cells

#### 6.2.1. Limited Persistence In Vivo

A major limitation of CAR-NK cell therapy is its limited persistence in vivo due to the lack of cytokine support. Although this reduces the risk of cytokine release syndrome, it limits the efficacy of CAR-NK cell therapy. A suitable solution is to engineer NK cells with transgenes that code for cytokines that are expressed on membranes or released constitutively [24]. Multiple studies have shown that NK cells incorporated with the IL-2/IL-15 transgene or CAR structures supplemented with IL-15 have improved proliferation and persistence, but do not lead to cytotoxicity [24,114]. Furthermore, inducing NK cells with a memory-like phenotype by briefly activating them with a cytokine cocktail of IL-12, IL-15 and IL-18 can lead to cytokine-induced memory-like NK cells, which show enhanced responses against NK-resistant B-cell lymphoma [24,115]. CAR-NK cells also face similar limitations to CAR-T cells such as trafficking to tumour tissue, tumour heterogeneity and an immunosuppressive microenvironment. Multiple strategies to overcome these limitations have been extensively reviewed in previous articles [24,27].

#### 6.2.2. The Advances of the CAR Molecule

A total of four generations of CARs has been researched and developed. Table 5 gives an overview of each of the generations of CARs. The basis of the CAR structure requires the TCR’s intracellular CD3zeta signalling domain [1]. Additional exogenous cytokine IL-2 was required because the first generation of CAR-T cells could not release enough of it, causing inhibited cell proliferation that leads to limited persistence in vivo [13,116,117]. In the second generation, the addition of the co-stimulatory domain enhanced cell multiplication and cytotoxicity and the antitumour response was sustainable in vivo [101]. The co-stimulatory domain functions to provide a second signal that promotes cytokine synthesis to complete the activation of T-cells [117]. Third-generation CAR involves combining multiple signalling and co-stimulatory domains in order to enhance the CAR-T cells’ function [118]. The fourth generation of CAR was built by adding cytokine IL-12 to the base of the second generation’s construct, and this redirected the function of T-cells for universal cytokine killing (TRUCK) [9,119]. This enables cytokines and chemicals to be released to improve tumour cytotoxicity in the immunosuppressive tumour microenvironment [9]. Besides T-cell activation, innate immune cells are also attracted to eliminate antigen-negative tumour cells [119]. As mentioned, CAR structures based on T-cell receptor constructs are also functional in NK cells, but research on CARs that incorporate the activating receptor of NK cell and DNAX-activation proteins with the CD3zeta chain showed superior CAR-NK cell capabilities [11].

## 7. Applications of MSC in CAR-T/NK Cell Immunotherapy

CAR-immune cell therapies face many challenges, and research into innovative ways to overcome these adversities is underway. The use of MSCs is favourable due to their unique characteristics. There are currently no listed clinical trials on clinicaltrials.gov involving MSC in CAR-related immunotherapy. However, various articles on preclinical studies have shown how MSC and CAR have an effective synergic effect in immunotherapy. This is still a fairly new therapeutic approach involving two different cellular immunotherapy interventions. There are currently only a few preclinical trials that involve MSC-assisted CAR-T cell immunotherapy or engineered MSCs that express CAR.

### 7.1. Modifications of MSCs to Improve Its Efficacy in Anti-Tumour Activity or in Assisting CAR-T Cell Therapy

#### 7.1.1. Preclinical Trials on CAR-Expressing MSCs

##### Glioblastoma

There are many challenges in treating glioblastoma (GBM), which is the most common primary malignant brain tumour [120]. The homing ability of MSCs play a vital role in this study. MSCs can home to GBM and not healthy brain cells, hence it serves as a tumour-specific drug-delivery system [121]. The MSCs are used to deliver a pro-apoptotic agent, the tumour necrosis factor-related apoptosis-inducing ligand (TRAIL). Besides that, a bi-functional MSC is engineered so that it delivers TRAIL and expresses the anti-GD2 CAR in order to enhance its immunoselective recognition ability towards GD2-positive tumours [122]. GD2 is an attractive antigen target due to its high level of expression in GBM [122,123]. The anti-tumour abilities were enhanced with the improved site-specific targeting based on multiple in vitro assays, and this may promise the prolonged retention of MSCs [122]. Previous studies have also documented the ability of MSCs as a delivery system for TRAIL. The systematic delivery of MSC-hTRAIL was able to prolong the survival of brainstem glioma-bearing mice. Its efficiency in delivering TRAIL against brainstem gliomas in vivo was shown to be successful [124].

##### Ewing’s Sarcoma

A recent study on Ewing’s sarcoma (ES), which is an aggressive type of cancer that affects children and young adults, shows the efficacy of modified MSCs. Due to ES’s metastatic characteristics, it is necessary to support MSC targeting to multiple remote sites. Hence, bi-functional MSCs that express TRAIL and truncated GD2-specific CAR were engineered. These MSCs were able to recognise and kill tumour cells in vitro. Furthermore, the anti-GD2 CAR improved tumour targeting and the persistence of bi-functional MSCs in vivo for lung ES but not liver ES [125].

#### 7.1.2. Preclinical Trials on MSC Assisted CAR-T Cell Therapies

##### Colorectal Cancer

MSCs are also engineered to release the cytokines IL7 and IL12 to promote homeostatic expansion and Th1 polarisation in the treatment of colorectal cancer. Thus, this is an example in which MSCs are used as vehicles to deliver immune-modulatory proteins to the target tumour site. In the study, bone-marrow-derived MSCs were engineered to release IL7, IL12 and both IL7 and IL12. They were able to sustain and enhance the anti-tumour activity of CAR-T cells against colorectal cancer. IL7 can promote homeostatic expansion and sustain the memory cell function of T-cells [126]. IL12 can induce a protective Th1 response and prevent Th2 polarisation of T-cells [127] as well as activate an innate immune response to eliminate cancer cells invisible to CAR-T cells [128]. The impact of MSCs also depends on the MSC-to-CAR-T-cell ratio. To summarise the study, the report shows that IL7 improves the persistence of CAR-T cells and other cytotoxic lymphocytes in peripheral blood and tumour tissue, whereas IL12 can enhance anti-tumour activity. Hence, this engineered MSC can work as an off-the-shelf cell therapy additive. This is one of the first studies that involve engineering MSCs to secrete cytokines to treat colorectal cancer [8].

##### Lung Cancer

McKenna et al. [129] worked on the delivery of oncolytic immunotherapy with engineered adenoviruses (OAd) to solid tumours by using MSCs as vehicles. The MSC systematically transported the OAd and a helper-dependent Ad (HDad), as a binary vector (combinatorial Ad vector [Cad]) that expresses IL12 and checkpoint PD-L1 blockers. Functional viruses are then produced to infect and kill lung tumour cells in addition to activating HER2-specific CAR-T cell anti-tumour activity through the release of IL-12 and PD-L1 blockers. 3D tumour spheroids were eliminated in vitro. The in vivo experiment showed growth suppression of two orthotopic lung cancer tumours. It is notable that with CAd-MSCs, the number of human T-cells was increased in vivo compared to the CAR-T-cell-only treatment. Their polyfunctional cytokine secretion abilities were also improved. This study shows how MSCs can help deliver oncolytic virotherapy to disrupt the TME and activate cancer cell lysis while stimulating CAR-T cell anti-tumour activities [129].

## 8. Advances and Strategies to Improve Overall MSC Efficacy as Vectors

Researchers have been exploring how MSCs are able to migrate to tumour sites across the endothelium. It is suggested that the damaged tissue express specific receptors or ligands that stimulate the trafficking, adhesion, recruitment and extravasation of MSCs to damaged and inflamed sites, which is similar to the mechanism of leukocyte homing. A more likely reason for specific migration is that tumours secrete chemotactic gradients so that MSCs can home to them. MSC derived from different sources have variable efficacy and efficiency as transporters. The most abundant source of MSCs would be bone marrow and adipose tissue, as these tissues have a great amount of MSCs and are easier to collect [130].

As a drug delivery vehicle, MSCs have been engineered to transport a variety of therapeutic agents for cancer treatment. These are apoptosis-inducing agents such as TRAIL, oncolytic viruses such as adenovirus and measles virus, tumour/tissue-specific prodrugs such as CD+5-5-FU, immunomodulatory agents such as IL12 and CXCL1, as well as cytotoxic chemotherapy such as paclitaxel and doxorubicin. It is evident that there are challenges when delivering anti-tumour therapeutics to non-target tissues and the threat of possible toxicities; hence, it is necessary to improve their tumour-specific targeting [131]. It is also notable that the dose of MSCs administered and the timing of administration matters in the promotion or inhibition of tumour growth [132].

MSCs infected with oncolytic adeno or measles viruses that can selectively replicate in tumour cells enhances the anti-tumour effects. MSCs can also be engineered to express prodrugs such as cytosine deaminase (CD) or herpes simplex virus-thymidine kinase (Hsv-TK). This converts inactive administered substrates to be activated as cytotoxic metabolites [131]. MSCs are also engineered to introduce various immunomodulatory proteins such as chemokines and interleukins. MSCs that deliver TRAIL as an anti-tumour strategy has been of great interest. A phase I/II study of MSC-TRAIL together with cisplatin and pemetrexed in non-small cell lung cancer has been carried out on patients (TACTICAL trial) [NCT03298763] [133,134]. Another way to apply MSCs in cancer treatment is by local delivery of MSCs directly to the target site. This reduces obstacles regarding homing and has a higher degree of overall safety [131].

In order to enhance homing, the lack of chemokine and adhesion molecules (PSGL-1, CXCR4 and E-selectin ligands) must be resolved. The CD44 expressed on MSCs are modified to HCELL (hematopoietic cell E-/L-selectin ligand) through the introduction of the enzyme fucosyltransferases to modify sugar moieties of the protein, allowing for better homing to the bone marrow [135]. It is also shown that CD44 expression can be increased, and subsequent homing can be enhanced via cultures on hyaluronic acid-coated plates in combination with the fucosylation strategy [136]. Genetic modification to express the fucosyltransferase in MSCs to convert CD44 to HCELL have also been studied [137]. Another approach that increased homing to gamma-irradiation-induced inflammation is using a transient multiplex cell-engineering strategy that combines homing modification, PSGL-1 and SLeX with the delivery of IL10 [138]. Furthermore, by using high-throughput screens, it is documented that small molecules can upregulate homing ligands such as ICAM-1 [139].

## 9. Conclusions and Perspectives

With the utilisation of CARs, T/NK cell immunotherapies can be directed towards specific tumour cells. The potential of CARs can be maximised by identifying the optimal tumour-associated antigens. However, CAR-based immunotherapies come with both advantages and limitations. These limitations, such as trafficking, immunosuppressive environment and limited persistence, can be overcome with the application of MSC. MSC’s diverse potential, from delivering various anti-tumour agents including oncolytic viruses and tumour-specific prodrugs to introducing immunomodulatory proteins such as chemokines and interleukins, not only enhances the therapeutic efficacy of CAR-related immunotherapy but also provides a promising future for solid tumour therapy. As such, suitable strategies to maximise the synergistic effects of both CAR and MSC by either using MSCs as a supplementary intervention to deliver molecules and assist in CAR-based immunotherapies or by modifying MSCs to express CARs as a directed therapy should be investigated further. In addition, the effects and mechanism of MSC in adoptive T/NK cell therapy would provide crucial early insights into the potential of MSC in CAR-T/NK cell immunotherapy.

## Figures and Tables

**Table 1:** Differences between CAR-T and CAR-NK cells [9,10,13,23,24,27,31].

Differences	CAR-T Cells	CAR-NK Cells
Intrinsic immune cell	T cells	Natural killer (NK) cells Immortalised human NK cell lines
Source of immune cell	Peripheral blood of patient	Peripheral blood from donor Umbilical cord blood Differentiated pluripotent stem cells
Surface expression of immune cell	T cell receptor (TCR), CD3	CD56, CD16
Shelf-life	Long-lived	Short-lived
Antigen recognition	Require prior antigen recognition	Do not require prior priming with antigen
Immune mechanisms	Stimulate apoptosis by activating the apoptotic signalling pathways within the cancer cells Cytokines enhance tumour clearance	Eliminate cancer cells via ADCC due to CD16 expression Induce apoptosis of the tumour cells by secreting tumour necrosis factor (TNF) Produce interferon-gamma upon engagement
Intrinsic cells that are reprogrammed	CD4+, CD8+	NK-92 cell (cell line) CD16+ CD56 dim (peripheral blood) CD16-CD56 bright (lymphoid tissues)
Receptor activated		NKG2D, NKG2C, NKp44, KIR
Co-stimulatory domain for specific CAR structures	CD28, CD137 (4-1BB), CD27, CD40, CD134	DAP10, DAP12, 2B4
Potential side effects	Cytokine release syndrome (CRS) Immune effector cell-associated neurotoxicity syndrome (ICANS) Risk of graft versus host disease (GVHD) due to allogenic source Tumour lysis syndrome Neurotoxicity On-target off-tumour toxicity Oncogenic insertional mutagenesis	Lack evidence of serious toxicities such as CRS and ICANS Does not induce graft versus host disease (GVHD)

**Table 2:** Similarities of CAR-T and CAR-NK cells [1,9,10,11,13,24,26,27].

Similarities of CAR-T and CAR-NK Cells
Derived from immune cells that are genetically engineered to express CARs
Require expansion and activation prior to infusion
Exhibit tumour cytotoxicity by releasing granzyme and perforin
Similar production protocols
Commonly used co-receptors: CD28, CD3z and 4-1BB
Independent of MHC
Utilise four generations of CARs and specific signalling/co-stimulatory domains
Common challenges: Trafficking to tumour sites, the immunosuppressive tumour environment, which is rich in immunosuppressive cytokines and metabolites
Amplification of the cytotoxicity activity via single-chain fragment variable (scFv) binding to respective tumour-associated antigen
Cytokines and chemokines release upon activation. (IFN-γ, TNF-α, IL2, IL6, IL12, IL21)

**Table 3:** An overview of different target antigens and the efficacy of modified CAR cells for non-solid tumour malignancies for CAR-T and CAR-NK cell therapy. Solid Tumour: Solid mass of cancer cells, grow in organ systems and can occur anywhere in the body, shows great level of antigen heterogeneity.

Disease	CAR-T Cell Therapy	CAR-NK Cell Therapy
**Ovarian cancer**	Targets: Tumour-associated glycoprotein 72 (TAG72), MUC16, Her2/neu, Mesothelin, 5T4, αFR TAG72-specific CAR-T cell (mice model): Cytotoxicity potential, cytokine production against OC cell lines and primary OC cells, reduced tumour growth, extended viability, reduced expression in early recurrent tumours [48] MUC16-specific CAR-T cell (in vitro, mice model): Efficient cytolytic activity, reduced OC progression, eradication of cancer cells [49] Mesothelin-specific CAR-T cell (in vitro, mice model): Inhibited tumour growth, improved survival, prolonged disease control, minimal off target toxicities [50] 5T4-specific CAR-T cell (mice model): Cytokine and IFNγ production, significant anti-tumour effects in SKOV-3 and OVCAR-3, good safety profile [51,52]	Targets: Mesothelin, CD24, αFR, CD19 Mesothelin-specific CAR-NK92 cell (in vitro, mice model): Kills cancer cells specifically in OVCAR-3 and SKOV-3, stronger cytokine production than CD19 CAR-NK/parental NK92 cells, effective tumour elimination in vivo, prolonged survival [53] CD24-specific CAR-NK92 cell (in vitro): Great cytotoxicity against CD24 positive OC cell lines and primary OC cells [54] αFR-specific CAR-NK92 cell (in vitro, mice model): High cytotoxicity and proliferation, low antigen-induced apoptosis, eliminate cancer cells, prolong survival [55]
**Breast cancer**	Targets: tMUC1, NKG2D ligands, HER2(ERBB2), mesothelin MUC28z CAR-T cells (mice model): Recognition for tMUC1 on TNBC cells, increased production of granzyme B, IFNgamma and Th1, reduced tumour growth, minimal off target toxicities [56] NKG2D CAR-redirected T-cells (in vitro, mice model): Effective recognition, elimination of TNBC cells, persistence of T-cell, deuteriation of TNBC, co-stimulation is necessary [57] HER2-specific CAR-T cells (in vitro): Significant cytotoxicity, apoptosis cell death in SKBR3, no toxicities in phase I/II clinical trials [58,59]	Targets: Tissue factor (TF), EGFR, HER2 TF-specific CAR-NK cell (in vitro, mice model): Kill TNBC cells, enhanced efficacy with L-ICON ADCC, effective anti-tumour activity [60] EGFR-specific CAR-NK cell (in vitro, mice model): Lysis of TNBC cells, inhibited CLDX and PDX tumours [61] HER2-specific CAR-NK92 cell (in vitro, mice model): Lysis and kill tumour cells, preserved anti-tumour activity in vivo [62]
**Lung cancer**	Targets: ROR1, EGFRvIII, mesothelin, EphA2, PSCA, mucin-1, PD-L1, delta-like 3 (DLL3) ROR1-specific CAR-T cell (3D tumour models) Deep penetration in tumour tissue, potent anti-tumour effects in A549 cell line (NSCLC), induced rapid apoptosis [63] EGFRvIII-CAR-T cell (in vitro, mice model) Cytokine production (perforin, granzyme B, IFNγ and TNFα), inhibits metastasis of A549-EGFRvIII cells, prolonged survival, no significant side effects [64] EphA2-specific CAR-T cell (in vitro, mice model): Tumour cell lysis in NSCLC, anti-tumour effects [65] Mesothelin-specific CAR-T cell (mice model): Slowed tumour growth of NSCLC, higher ability to kill tumour cells than T-cells [66] PSCA-redirected CAR-T cell and MUC1-redirected CAR-T cell (in vitro, mice model): Suppressed NSCLC tumour growth, eliminate tumour cells [67] PD-L1-specific CAR-T cell (in vitro, mice model): Antigen-specific activation, cytokine production, cytotoxic activity in NSCLC with PD-L1high and EGFR mutation [68] DLL3-targeted bispecific antibody and CAR-T cell (in vitro, mice model): Efficient anti-tumour effects in SCLC cell lines, suppressed tumour growth, improved efficacy of bispecific antibody with PD-1 inhibitory antibody [69]	Targets: CD19, CD20, EGFR, CD16, B7-H3, CD73 [70] B7-H3-specific CAR-NK92MI cell (in vitro, mice model): Enhanced cytotoxicity, increased cytokine secretion, limited tumour growth efficiently, prolonged survival significantly [71] NKG2D CAR-NK cell (in vitro, mice model): Mediated purinergic reprogramming, enhanced anti-tumour effects, improved intratumoural homing with CD73 blockade [72] NK92-CD16 cell (in vitro): Kills TKI-resistant NSCLC cells, enhanced cytotoxicity with cetuximab [73]
**Colorectal cancer**	Targets: NKG2D, CD133, GUCY2C, TAG72, DCLK1 NKG2D CAR-T cell (in vitro, mice model): Dose-dependent cytotoxicity, cytokine and chemokine secretion, suppressed tumour growth, reduced tumour size of HCT-116 cells, extend survival, minimal toxicity [74] CD133-specific CAR-T cell (phase I clinical study): Stable disease, elimination of tumour, controllable toxicities [44] GUCY2C-specific CAR -T cell (in vitro, mice model): Upregulation of activation marker, cytokine production, kill tumour cells, durable protection against metastasis [75] TAG-72 specific CAR-T cell (phase I clinical trial): 1st generation CAR showed low persistence, no off-tumour toxicity [76] DCLK1-specific CAR-T cell (in vitro, mice model): Cytotoxic activity targeting tumour stem cells, increases IFNγ secretion, reduced tumour growth [77]	Targets: NKG2D ligands, EpCAM, CEA EpCAM-specific CAR-NK92 cell (in vitro, mice model): Specific cytotoxicity and cytokine release, combination with regorafenib shows significance [78] CEA-specific CAR-NK92MI cell (in vitro): Lysis of CEA-expressing tumour cell lines that is CEA-dependent [79] NKG2D ligand-specific CAR-NK cell (pilot study): Decrease in ascites generation and number of tumour cells in ascites sample, rapid tumour regression in liver region [46]
**Pancreatic cancer**	Targets: alphavbeta6, B7-H3, PD-L1, CD24, PSCA, CEA, MUC-1, mesothelin, FAP, Her-2, CD-133 [1] αvβ6-specific CAR-T cell (in vitro, mice model): Coexpresssion of CXCR2, efficient migration to IL-8, significant anti-tumour activity, favourable toxicity [80] B7-H3-specific CAR-T cell (in vitro, mice model): Control tumour growth, minimal toxicity, 4-1BB co-stimulation promotes anti-tumour activity against PD-L1 expressing cancer cells [81] CD133-specific CAR-T cell (phase I clinical study): Stable disease, elimination of tumour, controllable toxicities [44] Mesothelin-specific CAR-T cell (phase I clinical study): Stable disease, expanded in blood, well tolerated, limited clinical activity [43]	Targets: FRα, death receptor 4/5 (DR4/5), MUC-1, PD-1, ROBO-1 FRα, DR4-specific CAR-NK cell (in vitro, mice model): Enhanced tumour-selective apoptosis [27,82] MUC1, PDL1-specific CAR-NK92 cell (phase I clinical trial): Lysis of cancer cells, stable disease, no severe cytokine storm/bone marrow suppression [47] ROBO1-specific CAR-NK92 cell (case study): Moderate in vitro efficacy, survival of the patient was 8 months [83]
**Glioblastoma**	Targets: B7-H3, CD147, GD2, MMP2, NKG2D ligands, CAIX, CD70, CSPG4, EphA2, TROP2, EGFRvIII B7-H3-specific CAR-T cell (in vitro, mice model): Significant anti-tumour effects, tumour regression [84] EGFRvIII-specific CAR-T cell (in vitro, mice model): Suppressed tumour growth of GL261/EGFRvIII GBM cells, eradicate tumour, inhibit antigenic heterogeneous GBM tumours [85] EGFRvIII-specific CAR-T cell (phase I pilot trial): Median survival 6.9 months, no clinically significant effect in patients [45] IL13Rα2-specific CAR-T cell (in vitro, mice model): Improved anti-tumour activity, improved T-cell persistence, intraventricular infusion shows benefits [86] HER2-specific CAR-T cell (in vitro): Potent cytotoxicity against U251 GBM cells, enhanced efficacy by anti-PD1 antibody [87]	Targets: IL-13Rα2, EGFRvIII, HER-2, EPHA2, CSPG4, CD133, CD70, EGFR, ErbB2 EGFRvIII-specific CAR-NK cell (in vitro, mice model): Designated with MR1.1-DAP12 and CXCR4, delay tumour growth, increased survival time, tumour remission [88] HER2-specific CAR NK92 cell (in vitro, mice model): Lysis of GBM cells, potent anti-tumour activity, protection against tumour rechallenge [89] wtEGFR and EGFRvIII-specific CAR-NK cell (in vitro, mice model): Enhanced cytolytic ability and IFNγ production, suppression of tumour growth, prolonged survival [90]

**Table 4:** CAR-T/NK cell therapies for B-cell malignancies, lymphoma and multiple myeloma. Non-solid tumour: Blood cancers such as leukaemia, lymphoma and myeloma; occur in the blood, bone marrow or lymph nodes.

Disease	CAR-T Cell Therapy	CAR-NK Cell Therapy
Acute lymphoblastic leukaemia (ALL)	Targets: CD19, CD20, CD22, CD123	Targets: NKG2D, CD19, FLT3
Chronic lymphoblastic leukaemia (CLL)	Targets: CD19, tyrsine-protein kinase transmembrane receptor	Targets: CD19, CD20
Lymphoma	Targets: CD19, CD30, CD20, κ-/λ-lightchain	Targets: CD20, CD4
Multiple myeloma	Targets: CD269, CD138	Targets: CD138, CS1

**Table 5:** A description of the generations of CARs [4].

Generation	Molecular Structure
First	CD3zeta signalling domain
Second	CD3zeta signalling domain + co-stimulatory domain CD28/CD137 (4-1BB)
Third	CD3zeta signalling + 2 different co-stimulatory domains CD28 and CD137 (4-1BB)
Fourth	CD3zeta signalling domain + co-stimulatory domain + chemicals/cytokines
